# Circulating tumor DNA in head and neck squamous cell carcinoma—current status and future prospects

**DOI:** 10.20935/acadonco7456

**Published:** 2024-12-19

**Authors:** Samuel Auger, Vasudha Mishra, Alka Singh, Yuxuan Miao, Nishant Agrawal, Evgeny Izumchenko

**Affiliations:** 1 Section of Otolaryngology-Head and Neck Surgery, University of Chicago, Chicago, IL 60637, USA.; 2 Department of Medicine, Section of Hematology and Oncology, University of Chicago, Chicago, IL 60637, USA.; 3 Ben May Department of Cancer Research, The University of Chicago, Chicago, IL 60637, USA.

**Keywords:** liquid biopsy, circulating tumor DNA, head and neck cancer

## Abstract

Squamous cell carcinoma (SCC) is the most common malignancy of the head and neck. Stagnating survival rates in recent decades, despite advances in the treatment paradigms, surveillance technologies, and multidisciplinary care, leave clinicians with a need for better options for screening, risk-stratifying, and monitoring patients. A growing proportion of patients with HPV-associated SCC have improved outcomes but continue to have a heterogenous response to treatment. Advances in the platforms and assays measuring circulating tumor DNA offer an opportunity to monitor disease status at the molecular level for both virally mediated and traditional risk-factor-driven SCC of the head and neck. This overview will discuss experimental, clinically used, and commercially available liquid biopsy platforms and their recent applications in patients with head and neck SCC malignancies.

## 1. Introduction

The application of diagnostic and surveillance technologies that use biomarkers in readily available bodily fluids such as blood and saliva has exploded in the last decade. These strategies are collectively referred to as “liquid biopsy” and have been met with significant academic and clinical enthusiasm for better screening, classifying, and/or surveilling patients with cancers, including head and neck squamous cell carcinoma (HNSCC). The continued refinement of technology for reliably measuring, with both high sensitivity and specificity, circulating tumor HPV-DNA (ctHPV-DNA) in the blood is poised to change the current paradigm of diagnosis, risk stratification, treatment, and surveillance of HPV-associated malignancies. Tumor-informed, DNA-mutation-based assays for HPV-negative tumors hold similar promise for patients with non-viral neoplasms of the head and neck. This field has quickly expanded to include biomarkers based on not only viral DNA but also a host of panels with high degrees of discrimination, including somatic mutations, exosomes, methylation status, circulating tumor cells, and non-genomic markers captured by metabolome, transcriptome, and microbiome analyses [1-6]. Some of these approaches center around the idea of identifying molecular residual disease (MRD), evidence of cancer-associated biomarkers, during or after definitive treatment. To be applied broadly, any diagnostic or surveillance strategy using a liquid biopsy approach must perform similarly to tissue biopsy and existing surveillance techniques regarding its sensitivity and specificity. Technologies are constantly evolving, and advances in platforms such as droplet digital PCR (ddPCR) and next-generation-sequencing-based assays (NGS) deliver consistent and reproducible results with the requisite high precision. Further refinement of these techniques and assays promises improved accuracy, speed, scalability, and affordability, allowing for their broad adoption in the domain of screening, surveillance, and subtyping of cancer patients.

The state of the field in 2022 was reviewed, with a comparison of the various techniques and platforms available for liquid biopsy [7]. Several groups have reviewed the literature on this topic, with emphasis on the growing implications for HPV-associated oropharyngeal cancer treatment [8-11], the use of methylation markers in oral cavity cancer [5], and summarizing the technical capabilities of these approaches [12]. Considering the rapid progress and expansion of the clinical application of circulating tumor DNA (ctDNA) and other liquid biopsies, this review will focus on the commercially available platforms, recent advances in the performance of ctDNA assays, and the clinical incorporation of ctDNA for both HPV-positive and HPV-negative HNSCC. Non-DNA markers will not be discussed. PubMed was queried for articles published between 2021 and 2024 containing the terms “circulating tumor”, “liquid biopsy”, “ctDNA”, and “head and neck”, yielding 727 articles to review. Articles that did not address cancers of the head and neck were excluded from the review. Manufacturer websites, clinical trial databases (clinicaltrials.gov), and institutional research websites were also interrogated for currently recruiting trials.

## 2. Liquid biopsy for viral-based biomarkers

### 2.1. ctDNA for HPV detection in OPSCC

Squamous cell carcinoma of the oropharynx (OPSCC) was traditionally seen as a disease afflicting those with heavy alcohol and tobacco use, with a natural progression and outcomes similar to those of squamous cell carcinoma of the oral cavity. Over the last decade, the discrete entity of HPV-associated OPSCC has emerged, now comprising 71% of all OPSCC cases in the United States [13]. Mechanistically, HPV proteins E6 and E7 degrade regulatory protein p53 and inactivate the retinoblastoma (Rb) pathway, allowing for unchecked tumor growth [14]. While HPV vaccination may hold the potential to curtail this epidemic and could prevent disease in younger cohorts, poor vaccine uptake will limit this effect, and the incidence of HPV-associated OPSCC is projected to rise until 2060 [15]. Ang et al. first described that HPV-associated OPSCC has markedly improved the clinical outcomes relative to those of non-HPV-associated OPSCC, with a 3-year survival of 82.4% vs 57.1%, respectively [16]. Based on these data and a confirmatory prospective analysis, HPV status was incorporated into the AJCC 8^th^ edition staging for oropharyngeal SCC [17]. Given the significant survival benefit and markedly improved prognosis of HPV-associated OPSCC, there have been efforts to de-escalate the treatment intensity and thus the acute and long-term sequelae associated with full-dose chemoradiation. Despite these efforts, the response to treatment remains heterogeneous and difficult to predict. Recurrence in these patients can be devastating, with high rates of failure in those receiving salvage treatment. Capturing HPV-DNA offers the opportunity to subtype, monitor for treatment response, provide guidance for response-adaptive treatment, and surveille for recurrence after the completion of therapy. There have been several studies evaluating the role of circulating HPV-DNA in early screening [18], aiding in diagnosis with indeterminate fine-needle aspiration results [19], and retrospectively evaluating patients with HPV-associated cancer for HPV signals prior to diagnosis [20]. Several prospective trials have validated multiple commercial and research ctHPV-DNA platforms as highly predictive of progression-free survival and in many cases detecting MRD and recurrence earlier than the standard of care [21-23].

### 2.2. Commercially available platforms for circulating HPV-DNA detection

While there is a variety of commercially available tests for detecting HPV-DNA or RNA in tissue, there are no FDA-approved liquid-biopsy-based assays for HPV-associated malignancies to help guide the treatment decisions in patients [24]. Some have been validated in the clinical setting and are available for clinicians to use in clinical trials and other institutional protocols. The majority of these platforms are currently used in the setting of OPSCC as a companion test to tissue biopsies [10]. As larger cohorts of patients are evaluated longitudinally using commercially available ctHPV-DNA detection methods, our understanding of their sensitivity and specificity in both a diagnostic and prognostic setting continues to improve.

One of the commercially available platforms for the detection of circulating HPV-DNA in head and neck cancers is the HPV-SEQ ctHPV-DNA test, a next-generation sequencing (NGS)-based technology developed by Sysmex Inostics for HPV16/18 detection and quantification in plasma. This test evaluates dynamic ctHPV-DNA changes in response to treatment for locally advanced HPV-associated oropharyngeal cancer. HPV-SEQ had a high clinical sensitivity of 97.6% in detecting HPV in pre-treatment baseline samples [25]. Further, NavDX, a droplet digital (ddPCR)-based platform for measuring the tumor-tissue-modified viral (TTMV)-HPV-DNA in plasma specimens, was developed by Naveris. Several studies have reported that measuring the TTMV score assists in diagnosis, response to treatment assessments, and long-term surveillance in HPV-driven oropharyngeal cancer patients [26, 27]. In a recent study, another high-performance ddPCR assay, termed ctDNA HPV16 Assessment using Multiple Probes, or CHAMP-16, was reported to accurately detect the circulating HPV-DNA in plasma with high sensitivity [28]. Recently, studies have been conducted to compare the performance of different HPV detection modalities (ddPCR, NGS, and qPCR) in blood or oral rinse samples [29]. For instance, it was reported that in plasma specimens, both NGS and ddPCR had a sensitivity of ~70% compared to that of ~20% for qPCR in HPV-DNA detection. However, for oral rinse samples, NGS had a better overall sensitivity of 75% compared to that of either ddPCR or qPCR [29]. Likewise, Brian et al recently conducted the largest single-institution analysis of ctHPV-DNA using a custom HPV hybrid-capture-based NGS assay (HPVseek), along with other blood-based tests, such as ddPCR, serology, and tissue whole-genome sequencing, demonstrating the high performance capabilities of HPVseek for the diagnosis and prognostication of HPV-positive OPSCC compared to those of ddPCR and E6 serology [30]. Furthermore, utilizing a meta-analysis approach, it has been shown that NGS-based testing may be the most sensitive approach to ctHPV-DNA detection, followed by ddPCR [31].

### 2.3. ctHPV-DNA in clinical trials

In addition to the tests mentioned above, there are many institutionally developed assays and platforms being applied in the context of clinical trials for HPV-mediated OPSCC. These tests are generally collected prospectively and evaluated retrospectively. While there are actively recruiting trials using ctHPV-DNA for the treatment stratification and decisions regarding adjuvant treatment, published data on this are limited [32]. For instance, Chen et al. conducted a pilot study (NCT05307939) of using ctHPV-DNA-based selection of patients for active postoperative surveillance. This arm of the study experienced a 25% (3/12) rate of gross disease recurrence with or before a rise in ctHPV-DNA and was closed, failing to meet the primary endpoint of >85% gross disease-free survival at one year post-treatment [32]. This study approach emphasizes the need for the inclusion of additional clinical factors in selecting patients for active surveillance and the initiation of delayed adjuvant therapy prior to recurrence. The best practices and guidelines for incorporating liquid-biopsy-based approaches into clinical decision-making need further exploration and integration into clinical trials and other prospective studies. Recent data regarding ctHPV-DNA in the context of risk stratification, dose-responsive treatment, and surveillance are reviewed below.

#### 2.3.1. Risk stratification and dose response

One of the most active fields of study is that of risk stratification for the purposes of treatment de-intensification. There are multiple trials using ctDNA alongside traditional imaging markers to capture the response to neoadjuvant therapy and stratify patients into treatment de-intensification groups. OPTIMA II is a stage II, open-label, non-randomized clinical trial evaluating the response to induction immunochemotherapy in advanced HPV+ OPSCC. Patients received neoadjuvant nivolumab plus nab-paclitaxel and carboplatin and were stratified into treatment arms based on their response on post-induction imaging. ctHPV-DNA was measured at several time points and found to correlate closely with the response to induction therapy, with clearance during induction associated with a significant improvement in 2-year progression free survival (PFS) [33]. Building on this trial design, the investigators used an arenavirus-based therapeutic vaccine (termed the HB-200 platform) in addition to chemotherapy in the neoadjuvant setting to induce a CD8+ response, utilizing ctDNA and imaging to stratify the patients into reduced or full-dose treatment arms. Eighty-one percent of the cases (17/21) had a deep response (>50% tumor shrinkage), and those stratified into the transoral robotic surgery arm had no viable tumors in the final pathology evaluations [34].

Cao et al. compared plasma ctHPV-DNA in stage III HPV-associated OPSCC patients to MRI and FDG-PET prior to definitive chemoradiation and after two weeks from the start of treatment. The authors found that low ctHPV-DNA at the start of treatment and an early increase in ctDNA at week 2 of treatment were predictive of freedom from progression during therapy (*p* < 0.002 and *p* < 0.005). The authors suggest that this increase during early treatment may represent tumor cell death and increased shedding as it responds to treatment. Clearance of ctHPV-DNA later in treatment did not show as strong an association, suggesting there may be a role of early ctDNA response in risk-stratifying further treatment in these patients [35]. Similarly, an analysis of 40 patients with HPV-associated OPSCC without distant metastasis showed that clearance of ctHPV-DNA after the start of treatment was associated with improved disease-free survival. The authors defined a favorable clearance profile as having >200 HPV copies/ml pre-treatment and demonstrating >95% clearance by day 28 of definitive chemoradiation (CRT). Patients without favorable clearance had a less predictable response to CRT, with 14% of the patients in this group having recurrence [36].

#### 2.3.2. Surveillance

With the exception of Epstein–Barr virus monitoring in nasopharyngeal carcinoma, the current National Comprehensive Cancer Network (NCCN) guidelines for the surveillance of head and neck cancer do not endorse or recommend the routine use of these tests and do not differentiate by HPV status or anatomic subsite [37]. Recommendations include a follow-up physical exam with a flexible endoscopy every 1–3 months for the first year, every 2–6 months for the second year, and so on. The follow-up interval is flexible and informed by the treatment response on imaging and examination. The use of ctHPV-DNA holds substantial potential for monitoring for early and occult recurrence long before it is clinically apparent. Early assays were limited by their sensitivity and unable to detect the low copy numbers of HPV-DNA required for surveillance, but modern ddPCR and NGS platforms for ctHPV-DNA quantification are capable of sensitivities ranging from 82% to 98% and specificities greater than 97% [11]. One retrospective analysis of a large cohort of 1076 patients with HPV-associated OPSCC reported that a post-treatment rise in ctHPV-DNA was detected in 59 patients prior to clinical evidence of recurrence, leading to further investigation and confirmation of disease relapse in 55 cases. Among patients without clinical evidence of disease, the positive predictive value (PPV) for a single plasma test 3 months after treatment completion was 93% [38]. In another recent report, Sanz-Garcia et al. explored the value of ctHPV-DNA in the context of pre- and post-definitive therapy for patients with high-risk, locally advanced OPSCC. Their work highlighted the higher sensitivity of HPV-seq over ddPCR for MRD detection in HPV-driven tumors and the better performance of RaDaR (Residual Disease and Recurrence), a proprietary, personalized, tumor-informed mutation detection assay developed by NeoGenomics, paired with HPV-seq over a tumor-naïve mutation detection assay (Cancer Personalized Profiling by Deep Sequencing, aka CAPP-seq) in terms of its sensitivity for MRD detection in patients who relapsed within 1 year [39].

Improved detection of recurrence, measured as the lead time from a positive plasma ctHPV-DNA test to confirmation with imaging or biopsy, offers an opportunity for intervention with salvage surgery, radiation, or systemic therapy. The lead times in the recent literature range from 47 days to 3 months [20, 21, 40, 41]. The data are not conclusive, however. A recent pilot trial utilizing ctHPV-DNA (NavDX) to guide adjuvant treatment closed early due to the failure of ctHPV-DNA to provide any lead time prior to 3 of the 12 patients developing gross recurrent disease [32]. Ling et al. recently reported the diagnostic and prognostic performance of a custom HPV-NGS liquid biopsy plasma-based assay (“HPV-DeepSeek”), citing its improved performance relative to that of ddPCR, HPV serology, and clinical work-up at the time of diagnosis with HPV+HNSCC. This assay demonstrated improved accuracy in the detection of prognostic features, including high-risk HPV16 SNPs, viral integration events, and PIK3CA mutations [42].

While plasma-based liquid biopsy tests are the primary mode being investigated, expanding efforts into saliva and urine are being explored. Saliva is readily available in most patients and has long been an attractive source for non-invasive testing. Early work investigating this approach found its limited utility in detecting ctHPV-DNA in OPSCC (40%) when compared to tumor-associated mutations [2]. A small contemporary feasibility pilot study, however, paired HPV-DNA with several gene mutations to detect the recurrence in 5/6 patients [43]. In a larger study of 233 patients (the DE-ESCALATE trial) with HPV-associated OPSCC, salivary rinse was combined with plasma-based HPV-DNA assays. The lead time from a positive assay to confirmed recurrence after treatment was 4 months, although saliva was not analyzed as an independent assay [44]. Recent work from the University of Michigan explored an alternative that may facilitate screening and surveillance. Leveraging ultra-short transrenal cell-free tumor DNA technology validated in urologic cancer, Bhambhani et al. developed an approach that could detect HPV-associated OPSCC in urine that was concordant with the available plasma assays and captured DNA missed by conventional approaches [45].

### 2.4. OPSCC trials in recruitment

There are several clinical trials in active recruitment seeking to evaluate various de-intensification/de-escalation treatment paradigms, screening protocols, and novel therapies for HPV-associated SCC of the oropharynx. Memorial Sloan Kettering researchers are recruiting for a large trial to evaluate the sensitivity of a commercially available ctHPV-DNA assay in screening adults with multiple sexual partners without previously diagnosed HPV-associated malignancy [46]. A group at MD Anderson is evaluating the role of adjuvant anti-PD1 immunotherapy in patients with HPV-associated OPSCC in clearing persistent viral MRD, improving long-term survival, and measuring the tolerability of treatment [47]. Our institution has employed ctHPV-DNA monitoring in de-intensification clinical trials for several years, with one ongoing prospective trial designed to evaluate the response of ctHPV-DNA using a NGS platform during induction chemotherapy and definitive CRT and throughout disease surveillance [33, 34, 48].

### 2.5. Cost benefits of ctHPV-OPSCC assessments

There is significant psychological and financial toxicity associated with the treatment of head and neck cancer, and the costs of these assays relative to that of the current modalities must be assessed [49]. One study performed a cross-sectional cost comparison of a ctDNA surveillance paradigm for HPV-OPSCC with the National Comprehensive Cancer Network (NCCN)-recommended and imaging-intensive schedule [50]. The authors estimated the costs of baseline surveillance, as well as that in the setting of equivocal and concerning results, with the ctDNA strategy, revealing a significant cost benefit. This was especially pronounced in equivocal and concerning diagnostic scenarios. In a similar study comparing the performance of ctHPV-DNA tests with that of regular office visits with flexible laryngoscopies and regular imaging studies, it was revealed that 72 imaging studies and 2198 in-office visits were required to detect one recurrence in HPV-associated OPSCC, suggesting a potential cost reduction of 42% if these were replaced with ctHPV-DNA surveillance [51]. With these advancements and potential decreases in cost, some oncologists have started to incorporate HPV-DNA monitoring into the treatment protocols for HPV-associated OPSCC. The resulting clinic data have refined our understanding and interpretation of the viral kinetics in the post-treatment period.

## 3. Circulating EBV-DNA in nasopharyngeal carcinoma

Nasopharyngeal carcinoma (NPC), prevalent in south-east Asia, is closely associated with Epstein–Barr virus (EBV) infection [52]. The 5-year survival rate of NPC patients with stage I disease is around 95% and drops to 60% for advanced-stage disease [53, 54]. Therefore, the identification of patients with early-stage disease is crucial to potentially improving disease prognosis and treatment outcomes. Over the past few years, several EBV detection methods have been developed for diagnosis, prognosis, and post-treatment surveillance in patients with NPC [55-58]. Circulating EBV DNA in plasma has been established as a biomarker of nasopharyngeal carcinoma with a sensitivity and specificity of ~97% and 98%, respectively [58, 59]. However, the major challenge associated with the early diagnosis of NPC is that the majority of these patients remain asymptomatic at the early stages of the disease and present at an advanced stage with local or distant metastasis. Several pre-clinical and clinical studies are focusing on exploring the utility of population-based screening for plasma EBV DNA as an efficient tool for the early diagnosis of NPC [60]. Moreover, the utility of ctEBV-DNA as a reliable marker for real-time risk recurrence determination has been explored recently. Lv et al. conducted the EP-SEASON study (NCT03855020) to investigate the longitudinal ctEBV-DNA atlas during sequential chemoradiotherapy in EBV-associated NPC and identified that the real-time recurrence risks of patients change synchronously with the ctEBV-DNA dynamics, thereby highlighting that tracking ctDNA longitudinally during treatment would add value to patient clinical management in terms of real-time recurrence risk forecasting [61].

### 3.1. Clinical trials on circulating EBV-DNA detection in nasopharyngeal carcinoma

While there is currently no FDA-approved test for the detection and quantification of ctEBV-DNA in body fluids, a series of clinical trials have reported the screening of plasma ctEBV-DNA as a promising biomarker for early diagnosis, prognosis, and post-treatment surveillance in nasopharyngeal carcinoma patients [62, 63]. One such clinical trial conducted a prospective screening of plasma from more than 20,000 participants and determined that screening for NPC using plasma EBV-DNA can help identify patients at earlier stages of the disease or even asymptomatic patients for improved survival [64]. Another clinical trial currently accruing patients in southern China will assess the levels of EBV antibodies and EBV-DNA in nasopharyngeal brushing and plasma in a population at high risk of NPC, measured to determine the most efficient method of NPC screening [65].

## 4. ctDNA for non-virally mediated HNSCC

While most of the advancements in the space of liquid biopsies in HNSCC have evaluated and refined the detection of ctHPV-DNA in HPV-associated squamous cell carcinoma of the oropharynx, technological breakthroughs in developing assays and tumor-specific, mutation-based biomarker panels may lead to personalized surveillance for non-HPV-associated head and neck malignancies.

### 4.1. Commercially available platforms and assay en route to commercialization

Exploring the use of mutation-based ctDNA to measure minimal residual disease in non-virally mediated head and neck cancers has recently gained broad interest and attention among researchers and clinicians, albeit with limited success. There is an unmet need for diagnostic tests that can predict the risk of recurrence in selecting patients for adjuvant therapy. Droplet Biosciences has introduced TruSight Oncology 500, an NGS assay based on a 500-gene panel for the detection of minimal residual disease in surgical drain fluid as a novel liquid biopsy analyte in HPV-negative head and neck cancer [66-68]. It has reported that increased ctDNA in patients’ lymph fluid is significantly associated with disease recurrence compared to that in patients with low or undetectable levels of lymph ctDNA. Furthermore, the Signatera assay developed by Natera utilizes 16-plex PCR to detect ctDNA in the plasma as an early indicator of disease progression or a favorable response during or post-treatment in HPV-negative HNSCC [69]. Using this tool, Hanna et al. found that pre-treatment mutational ctDNA was detectable in 86% of 116 patients with HPV-negative HNSCC. Moreover, patients with a positive assay after treatment had a significantly worse PFS (HR 7.33, *p* < 0.01), with a relatively poor 1-year overall survival (89.1% vs. 100%; HR: 7.33; *p* = 0.16) [70]. While the assays mentioned above focus on patients’ plasma, OrisDX, a startup founded by physicians and scientists from the University of Chicago and John Hopkins University, has recently developed an NGS method for saliva-based detection of cancer-driving somatic mutations for the early detection of HPV-negative oral cavity squamous cell carcinoma [1]. This salivary diagnostic platform couples high-throughput NGS with comprehensive computational algorithms to accurately detect oral-cancer-associated genetic biomarkers for early diagnosis of this disease. A summary table of the commercially available assays outlined in the text above, both viral and non-viral, and their characteristics is available in Table 1.

### 4.2. Non-HPV SCC ctDNA in clinical trials

In a study of 116 patients with non-HPV-associated cancer of the head and neck, whole-exome sequencing was performed on tissue samples, and personalized ctDNA panels were created using a commercially available 16-plex PCR product previously FDA-approved for breast, lung, colon, and bladder cancer. A total of 75% generated a detectable ctDNA-mutation-based signal post-treatment. Seventeen patients had persistently positive ctDNA values after the start of treatment, which was significantly associated with a worsened PFS when compared to those with negative ctDNA (HR: 7.33; 95% CI: 3.12–17.2; *p* < 0.001) [70]. Another example of using custom ctDNA panels for disease monitoring comes by way of the LIONESS study, which evaluated HPV-independent tumor-specific somatic mutation panels based on whole-exome sequencing of tumors (RaDaR) [71]. Each panel included 34–52 somatic variants and was able to detect the mutations at a reported variant allele frequency (VAF) below the advertised sensitivity of the assay. In a cohort of 17 patients, the assay demonstrated 95% sensitivity and 100% specificity, with ctDNA detected prior to recurrence in all cases that eventually recurred. The lead time of detectable ctDNA to recurrence ranged from 108 to 253 days. Recently, using custom ctDNA panels for the detection of MRD and the evaluation of potential tumor-informed ctDNA analysis for early molecular detection prior to clinically confirmed recurrence, Flash et al. conducted the LIONESS study. This trial assessed ctDNA for HPV-negative tumor-specific variants in plasma and saliva specimens from 76 patients with HNSCC utilizing whole-exome sequencing. In 617 longitudinally collected plasma samples, pre- and postoperatively, ctDNA was detected at a variant allele frequency (VAF) of 0.036%. A total of 34% of positive samples had ctDNA levels detected at a VAF of ≤0.01%. Additionally, increased plasma ctDNA levels were detected postoperatively in 96% of cases with confirmed clinical recurrence (21/22), with a median lead time of 160 days. Furthermore, ctDNA was also detected in the baseline saliva samples, displaying a 74% overlap with the corresponding plasma ctDNA profiles. The subgroup analysis of a multicenter randomized controlled trial (IMSTAR-HN) found that the individualized ctDNA panels of somatic mutations obtained via ddPCR of 19 patients with HPV-negative cancers were predictive of recurrence. After validating the assays against tumor sequencing, none of the patients with a full ctDNA response after treatment (n = 8) had recurrence after the two-year follow up. Of the four patients with persistent ctDNA, one experienced distant failure at 36 weeks, and another had local recurrence at 73 weeks [72, 73]. Some researchers are investigating assays that are agnostic of, but inclusive of, HPV status. A recent study evaluated the potential of a combined NGS-based, tumor-agnostic plasma ctDNA assay targeting 26 genes and HPV-16 in patients with locally advanced HNSCC within 12 weeks post-treatment. MRD was identified in 41% of the patients with detectable ctDNA at baseline [74]. There are also groups evaluating personalized tumor-based assays for monitoring for non-HPV-associated oropharyngeal cancer. The COMPLETE trial aims to evaluate 60 patients with HPV-independent OPSCC undergoing CRT at a single center using a macroscopic, microscopic, and molecular layer of analysis, with the goal of determining whether an early change in these metrics may predict overall treatment response and survival [75]. Macroscopically, diffusion-weighted MRI imaging was used to assess the relative change in the diffusion coefficient *D* at the primary tumor site after the initiation of treatment. Microscopically, the biopsies were irradiated with 5 gy ex vivo, and a radiosensitivity analysis was performed. Finally, an NGS ctDNA panel of eight somatic mutations will be used to analyze blood samples from pre-, during, and 3 months post- treatment. A summary of the clinical trials described above, including their clinical settings, outcome measures, and years of collection, is provided in Table 2.

## 5. Future directions

Tissue biopsy for diagnosis is unlikely to be replaced, but rather to be supplemented and complemented, by the techniques and assays outlined above. As the accuracy of tests and their cost come down, however, their broader availability may improve the care for patients with head and neck cancer in several ways. Further, the adoption of multi-cancer early detection tests will lead to new referral patterns and require head and neck oncology teams to navigate the diagnostic work-up and patient counseling [76].

The current surveillance paradigms rely on physical exams, flexible endoscopies, and imaging modalities for detecting recurrence.

Growing evidence increasingly supports the use of liquid biopsy to detect the recurrence of head and neck cancer. Tumor-specific, mutation-based, and viral-based ctDNA tests may all be incorporated into the best practices for surveillance [71, 72]. Additional evaluations of their cost-effectiveness in the surveillance setting may lead to the more judicious application of costly imaging exams in low-risk patients. A survey of physicians and patients found that 84% of patients favored using HPV-DNA to follow their disease status [77].

## 6. Conclusions

Circulating tumor DNA will continue to be incorporated into clinical trials and institutional protocols, informing the best practices for screening and risk-stratifying, adapting treatment for, detecting occult recurrence in, and caring for patients with cancer of the head and neck. Currently, the protocols for surveillance are not personalized to individualized risk factors and rely largely on clinicians’ judgments. ctDNA approaches offer improved lead times in the recognition of recurrence and the potential for earlier treatment. The optimal timing and sensitivity thresholds needed for the routine use of these assays in the clinical setting remain to be determined. Regardless of the timing of their broader adoption or incorporation into the guidelines, there will continue to be a significant amount of energy expended on and investment into these technologies, with rapid advancements in their sensitivity, cost, and availability to clinicians and patients who wish to use them.

## Figures and Tables

**Table 1 •:** Summary of commercially available platforms for ctDNA detection in head and neck cancers.

Commercial partner	Test/platform	Biofluid/analyte	Analytical technique	Clinical application	Sensitivity/specificity	Reference
Sysmex Inostics	HPV-SEQ	Plasma, cfHPV-DNA	NGS-based technology for HPV16/18 detection	Real-time HPV quantification that evaluates treatment response and dose de-escalation, reducing treatment-related toxicities with improved survival for HPV-driven head and neck cancers.	Sensitivity—97.6%	[24]
Naveris	NaVDx	Plasma, *TTMV-HPV cfDNA	Droplet digital PCR (ddPCR)-based technology for HPV detection	Offers rapid HPV detection with improved outcomes by assessing treatment response, monitoring recurrence, and identifying post-treatment residual disease.	Sensitivity—89%; specificity—97%	[26]
-	*CHAMP-16	Plasma, HPV ctDNA	High-performance droplet digital PCR (ddPCR) for HPV detection	High-performance and cost-effective HPV detection assay for screening and post-treatment surveillance in HPV+ve OPSCC.	Sensitivity—100%	[27]
-	HPVseek	Plasma, ctHPV-DNA	HPV whole-genome hybrid-capture-based NGS assay	Non-invasive test for the diagnosis and prognosis of HPV+ve OPSCC.	Sensitivity—99%; specificity—100%	[29]
Droplet Biosciences	TruSight Oncology 500	Surgical drain fluid/lymph fluid, cfDNA	NGS-based 500-gene panel testing	Detects minimal residual disease (MRD). Predicts recurrence in HPV-negative head and neck cancers. Potential to provide personal adjuvant treatment options.	Sensitivity—89%; specificity—67%	[65-67]
Natera	Signatera	Blood, ctDNA	16-plex-PCR-based technology for mutation detection	A highly sensitive and personalized MRD assay as an early indicator of relapse or favorable response in HPV-negative head and neck cancers.	Sensitivity—98%; specificity—99.5%	[68]
OrisDx	OrisDx	Saliva	NGS-based analysis of specific gene mutations	A non-invasive diagnostic platform for rapid and accurate detection of genetic biomarkers of diagnosis and disease progression in HPV-negative head and neck cancer.	Sensitivity—93%; specificity—99%	[1]

* TTMV—tumor-tissue-modified viral

* CHAMP-16—ctDNA HPV16 Assessment using Multiple Probes.

**Table 2 •:** Clinical trials with circulating tumor cell-based treatment decisions.

Clinical trial ID	Aim	Study design	Type of HNSC	Sample size	Status	Sample	Primary endpoints/major outcome	Year of study (start date–estimated year of completion)
NCT03107182 [32]	To determine the deep response rate and tolerability of the addition of neoadjuvant nivolumab to chemotherapy followed by response-adapted locoregional therapy (LRT) in patients with HPV+ OPC.	Interventional; phase 2 non-randomized clinical trial	HNSCC; HPV-related squamous cell carcinoma; human papillomavirus-positive oropharyngeal cancer (HPV+ OPC)	76 patients with locoregionally advanced HPV+ OPC	Completed	Blood	Tumor response rate, progression-free survival (PFS), and overall survival (OS); ctHPV-DNA clearance was significantly associated with improved PFS.	2017–2024
NCT05108870 [33]	To determine the safety, deep response, and de-escalation with HPV16-specific arenavirus-based immunotherapy (HB-200) plus chemotherapy followed by response-stratified de-intensification in HPV16+ oropharyngeal cancer.	Interventional; phase 1/2 randomized clinical trial	HNSCC; human papillomavirus-positive oropharyngeal squamous cell carcinoma (HPV+ OPC)	21 patients with non-metastatic HPV16+ OPC; for phase 2, estimated sample size = 98	Recruiting for phase II	Plasma and sputum	Safety/tolerability, tumor response rate, ctHPV-DNA dynamics, and HPV16-specific T-cell response. Neoadjuvant HB-200/chemotherapy was safe and feasible, with early efficacy signals.	2017–2026
NCT04572100 [47]	To evaluate dynamic changes in ctHPV-DNA during induction therapy, definitive (chemo)radiotherapy, and post-treatment surveillance in the context of risk and response-adaptive treatment for HPV + OPC.	Interventional, prospective, non-randomized, phase 1	Oropharyngeal squamous cell carcinoma	50 patients with locoregional HPV + OPC	Completed	Blood	ctHPV-DNA dynamics, tumor response rate, and overall survival. Correlation of quantitative ctHPV-DNA with radiographic response. Assessment of quantitative ctHPV-DNA represents a reliable. personalized, adaptive de-escalation strategy in HPV-associated OPC.	2020–2023
NCT02063399 [63]	To screen nasopharyngeal carcinoma using plasma Epstein–Barr virus DNA analysis.	Prospective study	Nasopharyngeal carcinoma	20,000 patients with nasopharyngeal carcinoma	Ongoing	Plasma	Sensitivity and specificity of plasma EBV DNA analysis were 97.1% and 98.6%, respectively.	2013–2023
NCT05447169 [64]	To evaluate the diagnostic accuracy of Epstein–Barr virus antibodies and Epstein–Barr virus DNA for nasopharyngeal carcinoma screening in the high-risk population.	Prospective study	Nasopharyngeal carcinoma	11,625 patients with nasopharyngeal carcinoma	Recruiting	Nasopharyngeal brushing and peripheral blood	Cancer screening; diagnostic test.	2022–2030
NCT03700905 [71]	To detect early relapse through monitoring of circulating cell-free DNA in patients with localized head and neck squamous cell carcinoma.	Multicenter, randomized clinical trial (IMSTAR-HN)	HNSCC	19 patients from the IMSTAR-HN trial	Active, not recruiting	Plasma	Cell-free DNA mutations after surgery identify patients at risk of early relapse; increasing variant allele frequencies precede clinical progression.	2018–2024
NL8458 [74]	To study the multilayered tumor landscape based on novel techniques, focusing on the macroscopic, microscopic, and molecular landscape to assess changes in the tumor landscape early during treatment.	Single-center, prospective observational study	HNSCC ; HPV-negative oropharyngeal squamous cell carcinoma (OPSCC)	60 HPV-negative patients with OPSCC	Pending	Blood	Assessment of early tumor response.	2020–2024
NCT05307939 [31]	To evaluate selective minimal-residual-disease-directed adjuvant radiation in human papilloma virus-associated oropharynx carcinoma.	Interventional; phase 2 non-randomized clinical trial	HNSCC; human papillomavirus-positive oropharyngeal cancer (HPV+ OPC)	30 HPV+ OPC patients	Recruiting	Blood	Assessment of pathologically confirmed progression-free survival and early disease recurrence following HPV ctDNA-directed active surveillance	2022–2025

HPV = human papilloma virus; OPC = oropharyngeal carcinoma; HNSCC = head and neck squamous cell carcinoma; PFS = progression-free survival; OS = overall survival; EBV = Epstein–Barr virus; ctDNA = circulating tumor DNA.

## Data Availability

Data supporting these findings are available within the article, at https://doi.org/10.20935/AcadOnco7456, or upon request.
